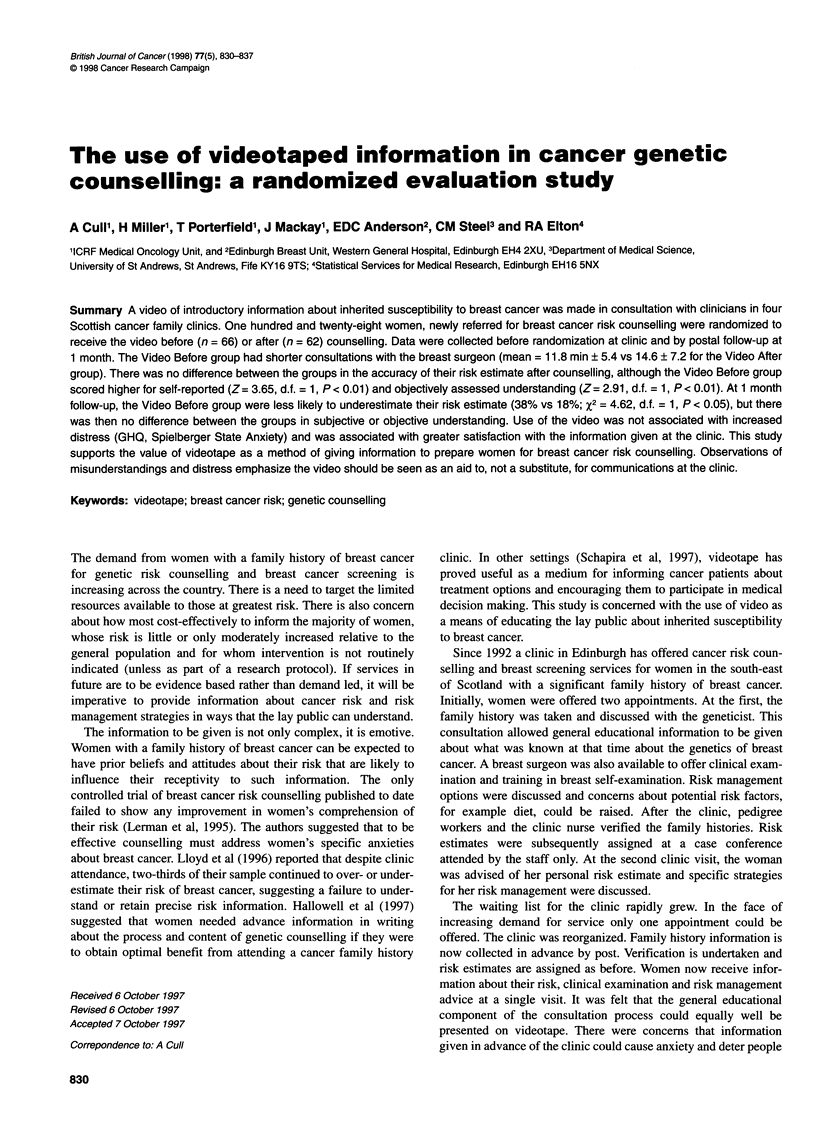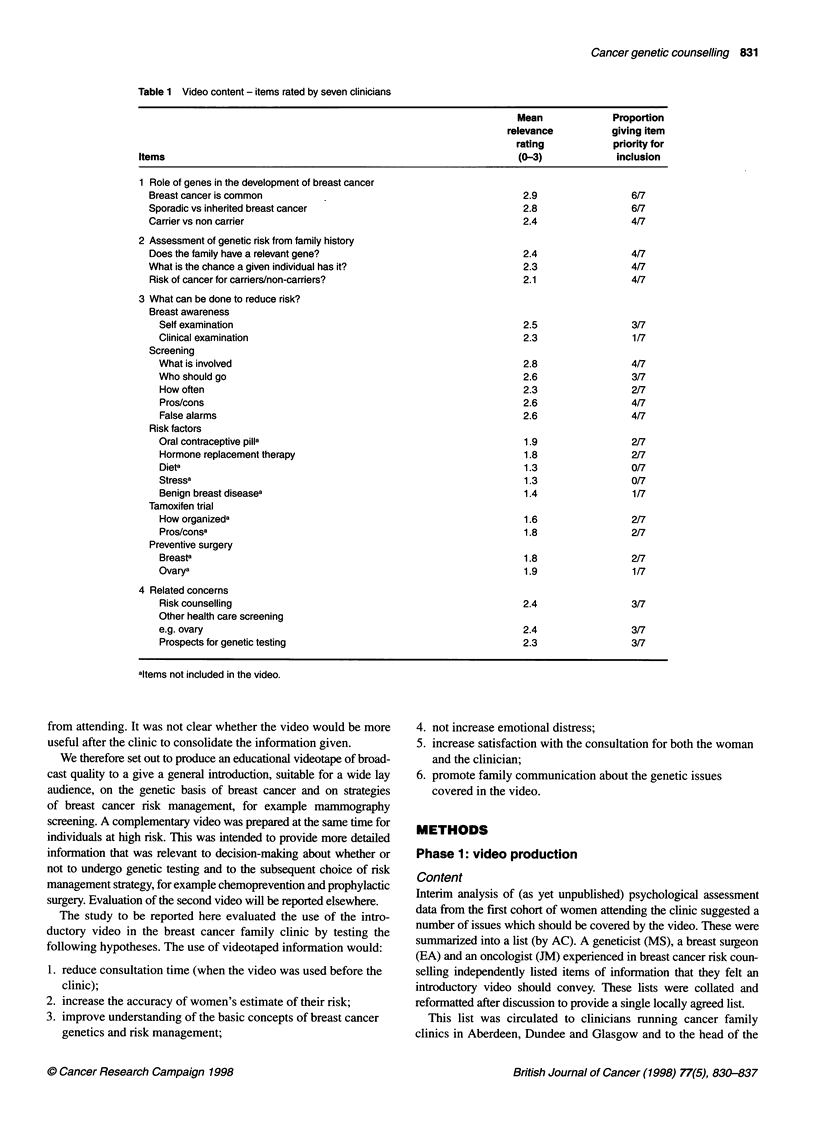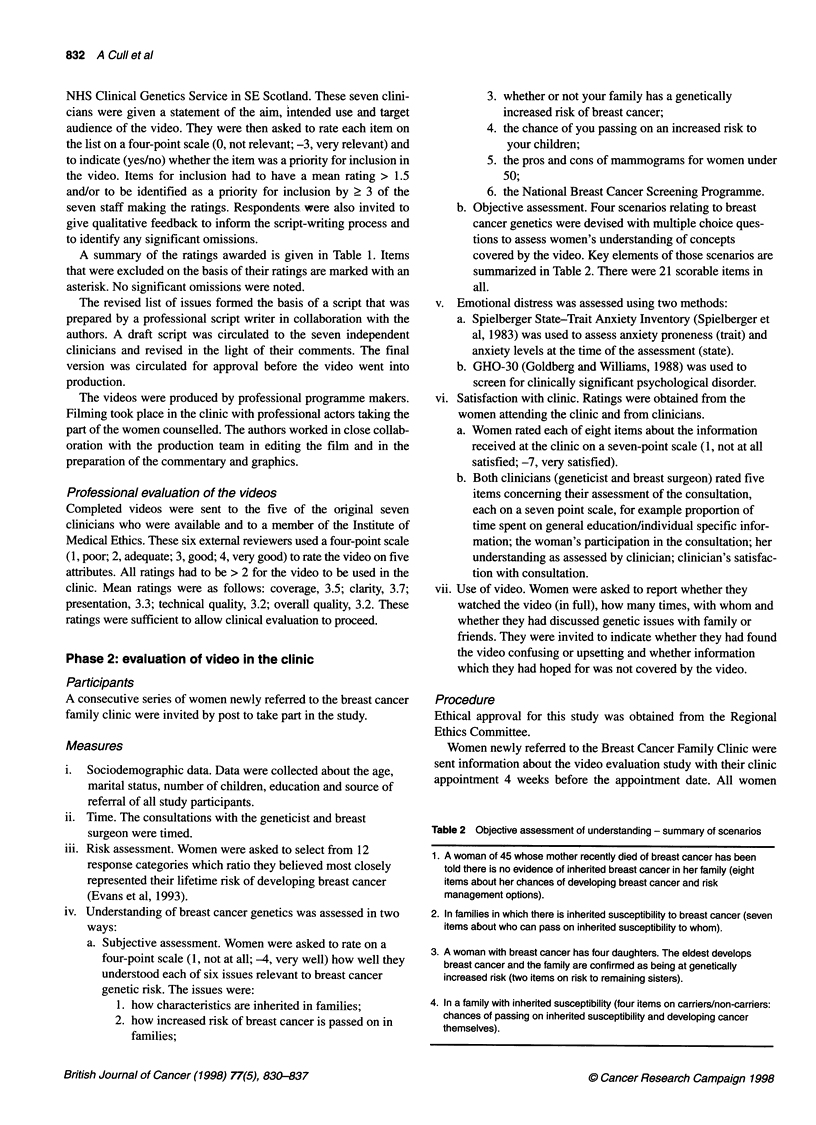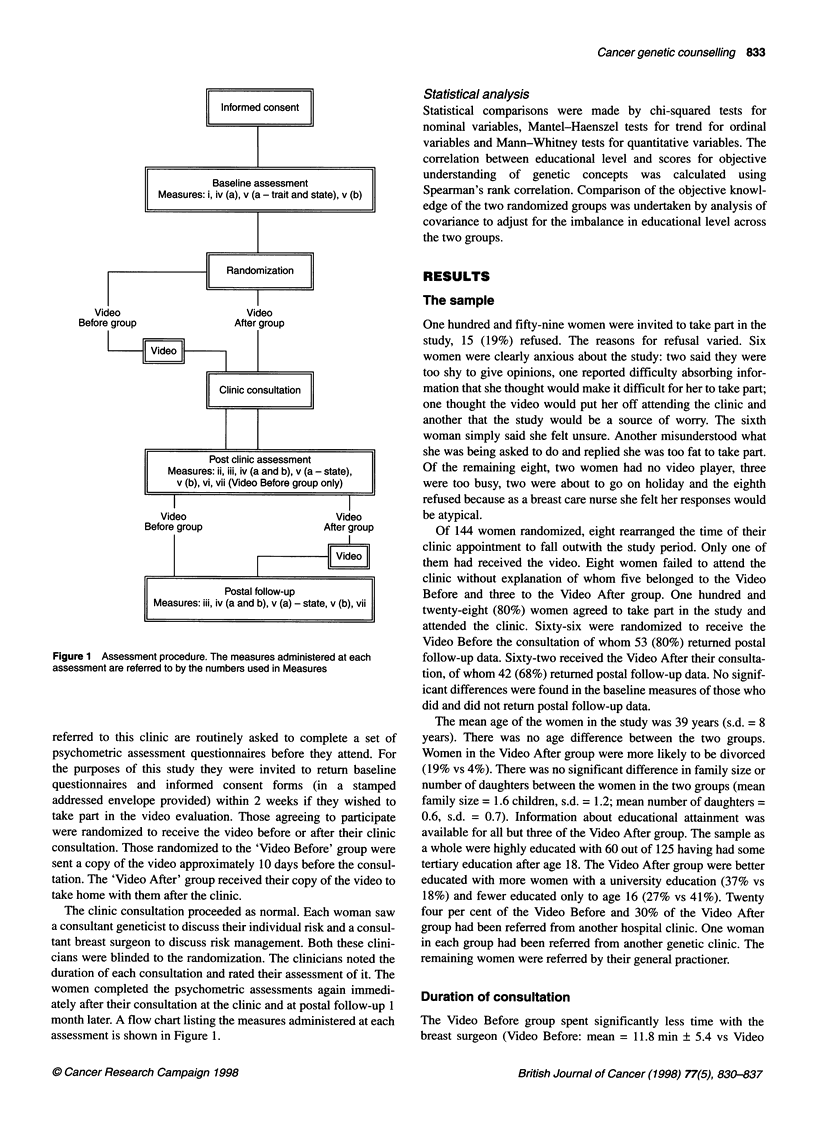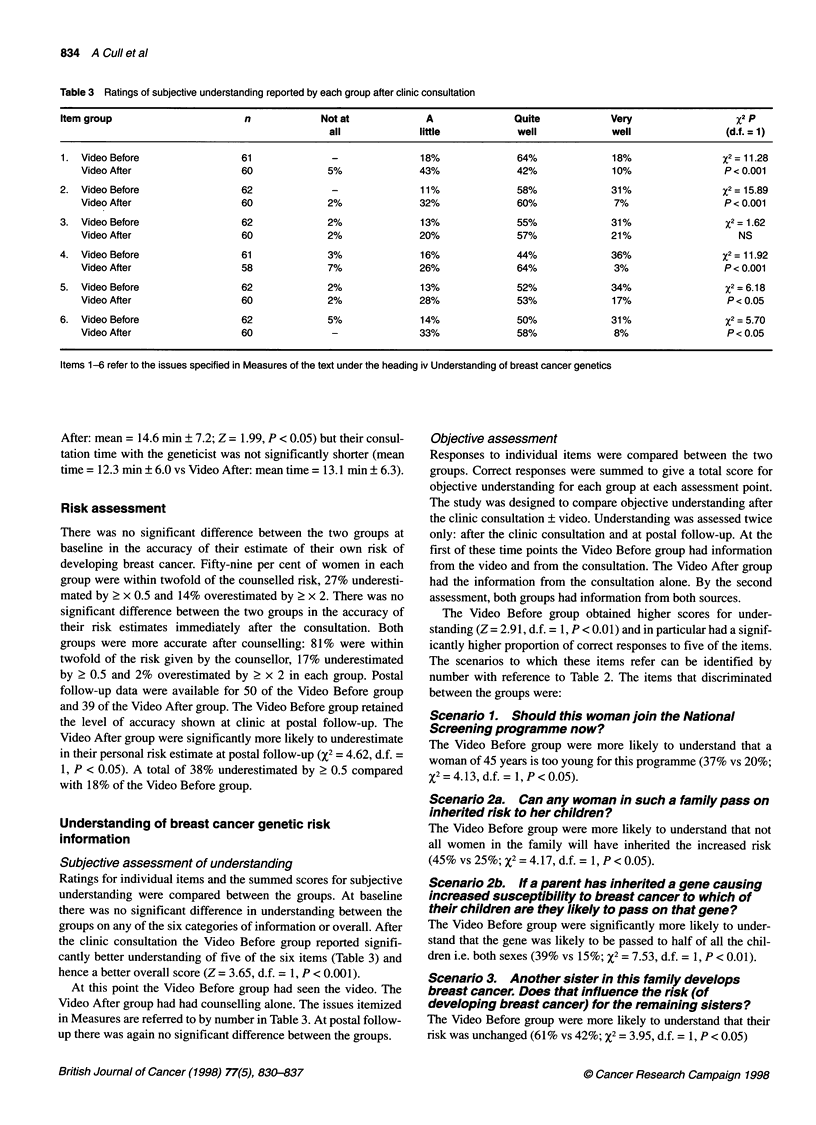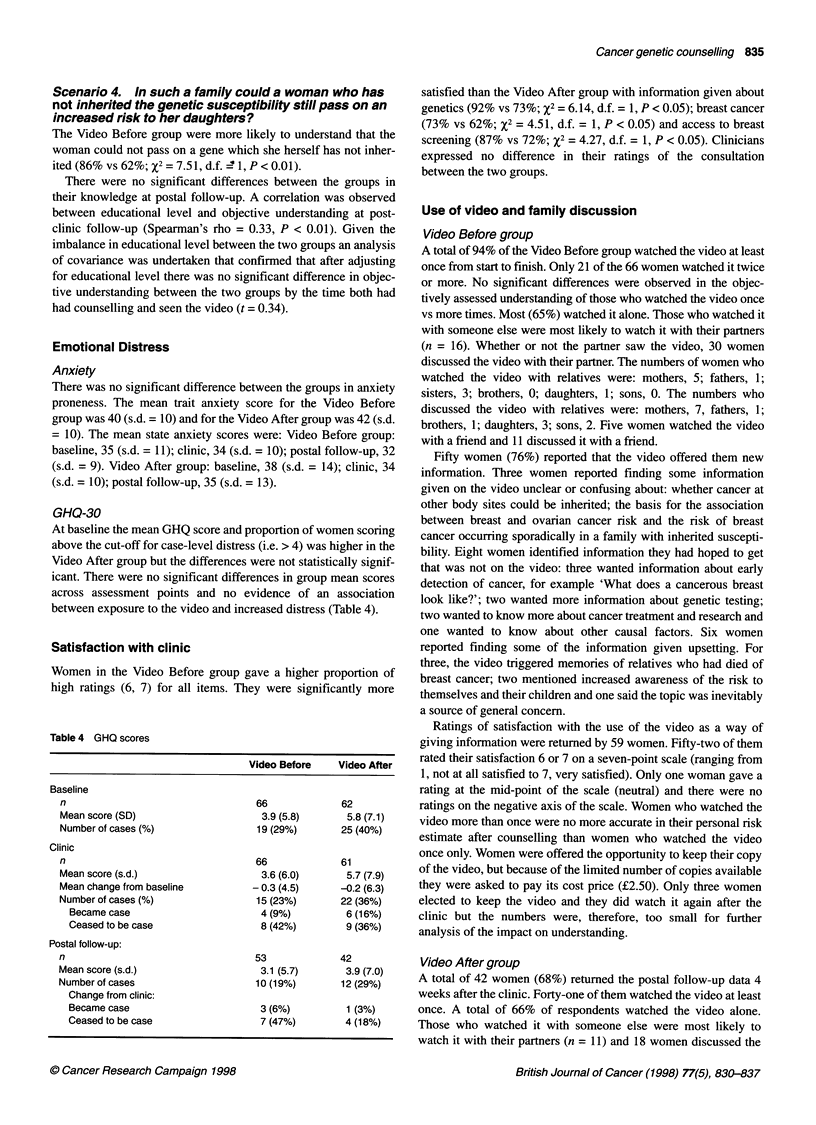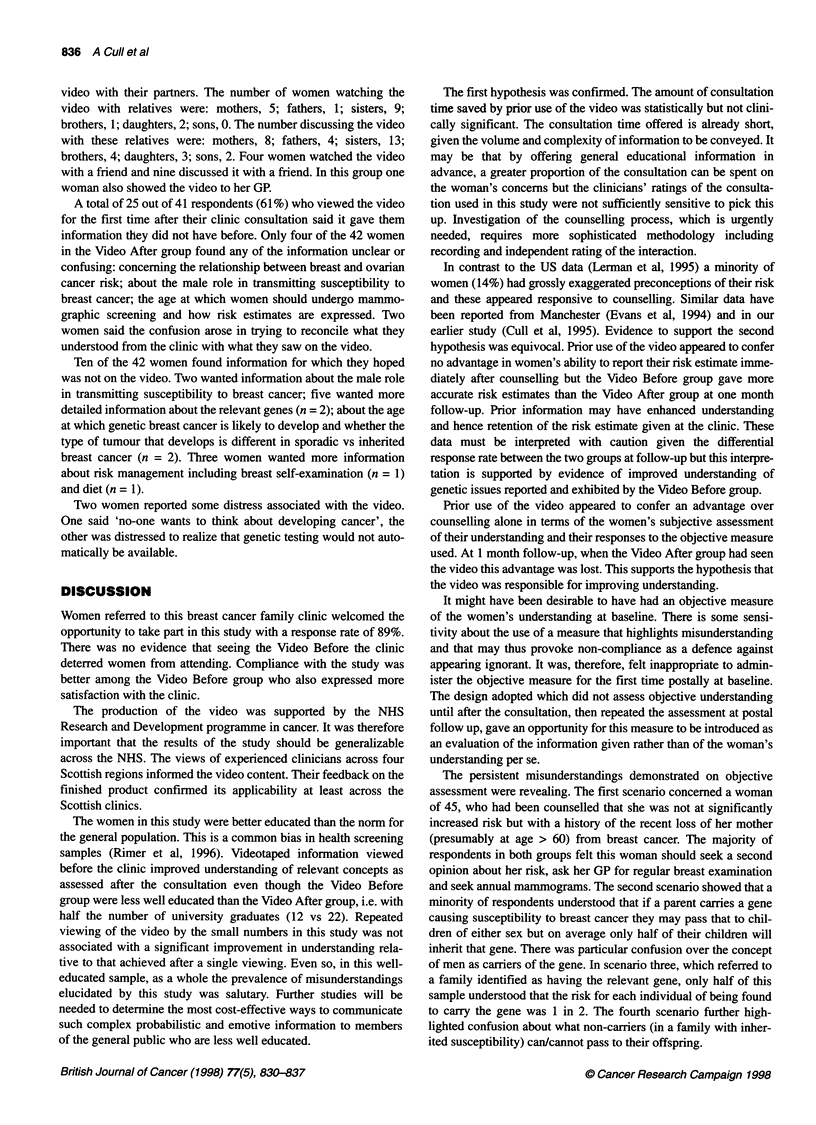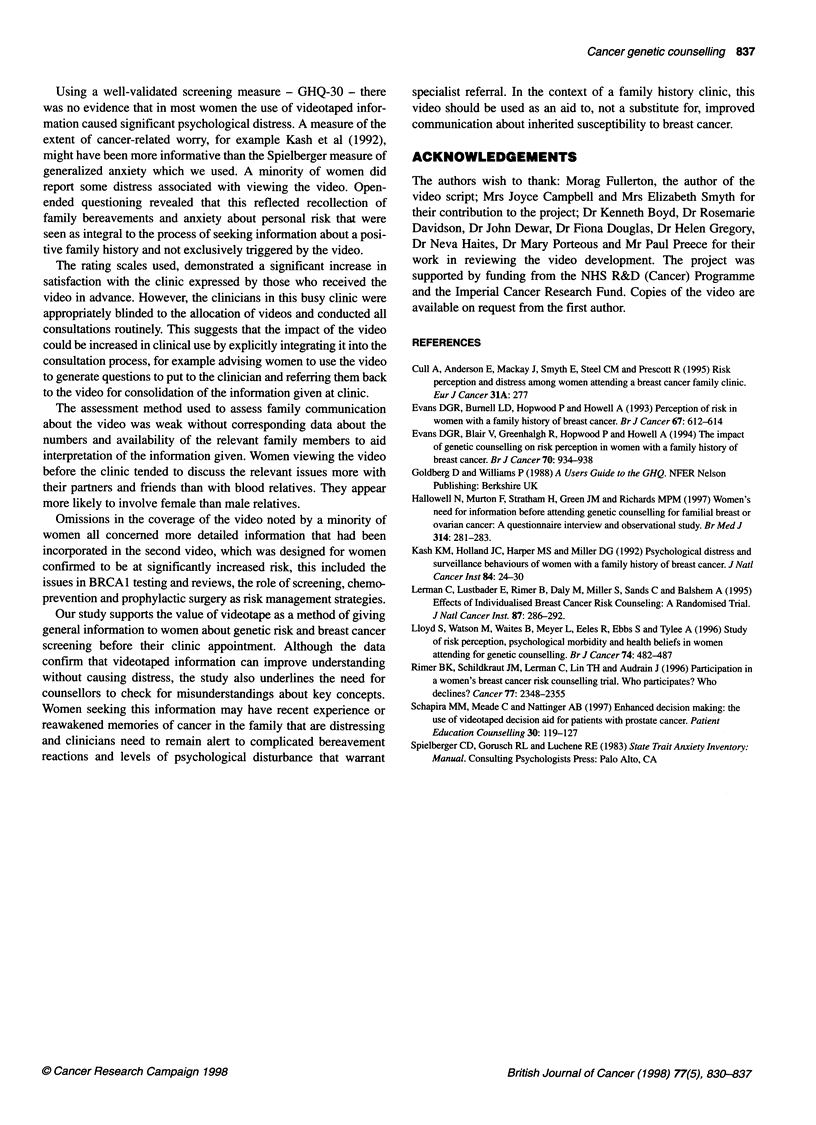# The use of videotaped information in cancer genetic counselling: a randomized evaluation study.

**DOI:** 10.1038/bjc.1998.135

**Published:** 1998-03

**Authors:** A. Cull, H. Miller, T. Porterfield, J. Mackay, E. D. Anderson, C. M. Steel, R. A. Elton

**Affiliations:** ICRF Medical Oncology Unit, Western General Hospital, Edinburgh.

## Abstract

A video of introductory information about inherited susceptibility to breast cancer was made in consultation with clinicians in four Scottish cancer family clinics. One hundred and twenty-eight women, newly referred for breast cancer risk counselling were randomized to receive the video before (n = 66) or after (n = 62) counselling. Data were collected before randomization at clinic and by postal follow-up at 1 month. The Video Before group had shorter consultations with the breast surgeon (mean = 11.8 min+/-5.4 vs 14.6+/-7.2 for the Video After group). There was no difference between the groups in the accuracy of their risk estimate after counselling, although the Video Before group scored higher for self-reported (Z= 3.65, d.f. = 1, P < 0.01) and objectively assessed understanding (Z= 2.91, d.f. = 1, P < 0.01). At 1 month follow-up, the Video Before group were less likely to underestimate their risk estimate (38% vs 18%; chi2 = 4.62, d.f. = 1, P< 0.05), but there was then no difference between the groups in subjective or objective understanding. Use of the video was not associated with increased distress (GHQ, Spielberger State Anxiety) and was associated with greater satisfaction with the information given at the clinic. This study supports the value of videotape as a method of giving information to prepare women for breast cancer risk counselling. Observations of misunderstandings and distress emphasize the video should be seen as an aid to, not a substitute, for communications at the clinic.


British Journal of Cancer (1998) 77(5), 830–837
© 1998 Cancer Research Campaign

### The use of videotaped information in cancer genetic counselling: a randomized evaluation study

**A Cull[1], H Miller[1], T Porterfield[1], J Mackay[1], EDC Anderson[2], CM Steel[3] and RA Elton[4]**

[1]ICRF Medical Oncology Unit, and [2]Edinburgh Breast Unit, Western General Hospital, Edinburgh EH4 2XU, [3]Department of Medical Science, University of St Andrews, St Andrews, Fife KY16 9TS; [4]Statistical Services for Medical Research, Edinburgh EH16 5NX

**Summary** A video of introductory information about inherited susceptibility to breast cancer was made in consultation with clinicians in four Scottish cancer family clinics. One hundred and twenty-eight women, newly referred for breast cancer risk counselling were randomized to receive the video before ($n$ = 66) or after ($n$ = 62) counselling. Data were collected before randomization at clinic and by postal follow-up at 1 month. The Video Before group had shorter consultations with the breast surgeon (mean = 11.8 min ± 5.4 vs 14.6 ± 7.2 for the Video After group). There was no difference between the groups in the accuracy of their risk estimate after counselling, although the Video Before group scored higher for self-reported ($Z$ = 3.65, d.f. = 1, $P$ < 0.01) and objectively assessed understanding ($Z$ = 2.91, d.f. = 1, $P$ < 0.01). At 1 month follow-up, the Video Before group were less likely to underestimate their risk estimate (38% vs 18%; $\chi^2$ = 4.62, d.f. = 1, $P$ < 0.05), but there was then no difference between the groups in subjective or objective understanding. Use of the video was not associated with increased distress (GHQ, Spielberger State Anxiety) and was associated with greater satisfaction with the information given at the clinic. This study supports the value of videotape as a method of giving information to prepare women for breast cancer risk counselling. Observations of misunderstandings and distress emphasize the video should be seen as an aid to, not a substitute, for communications at the clinic.

**Keywords:** videotape; breast cancer risk; genetic counselling

The demand from women with a family history of breast cancer for genetic risk counselling and breast cancer screening is increasing across the country. There is a need to target the limited resources available to those at greatest risk. There is also concern about how most cost-effectively to inform the majority of women, whose risk is little or only moderately increased relative to the general population and for whom intervention is not routinely indicated (unless as part of a research protocol). If services in future are to be evidence based rather than demand led, it will be imperative to provide information about cancer risk and risk management strategies in ways that the lay public can understand.

The information to be given is not only complex, it is emotive. Women with a family history of breast cancer can be expected to have prior beliefs and attitudes about their risk that are likely to influence their receptivity to such information. The only controlled trial of breast cancer risk counselling published to date failed to show any improvement in women's comprehension of their risk (Lerman et al, 1995). The authors suggested that to be effective counselling must address women's specific anxieties about breast cancer. Lloyd et al (1996) reported that despite clinic attendance, two-thirds of their sample continued to over- or under-estimate their risk of breast cancer, suggesting a failure to understand or retain precise risk information. Hallowell et al (1997) suggested that women needed advance information in writing about the process and content of genetic counselling if they were to obtain optimal benefit from attending a cancer family history clinic. In other settings (Schapira et al, 1997), videotape has proved useful as a medium for informing cancer patients about treatment options and encouraging them to participate in medical decision making. This study is concerned with the use of video as a means of educating the lay public about inherited susceptibility to breast cancer.

*Received 6 October 1997*
*Revised 6 October 1997*
*Accepted 7 October 1997*

*Correpondence to: A Cull*

Since 1992 a clinic in Edinburgh has offered cancer risk counselling and breast screening services for women in the south-east of Scotland with a significant family history of breast cancer. Initially, women were offered two appointments. At the first, the family history was taken and discussed with the geneticist. This consultation allowed general educational information to be given about what was known at that time about the genetics of breast cancer. A breast surgeon was also available to offer clinical examination and training in breast self-examination. Risk management options were discussed and concerns about potential risk factors, for example diet, could be raised. After the clinic, pedigree workers and the clinic nurse verified the family histories. Risk estimates were subsequently assigned at a case conference attended by the staff only. At the second clinic visit, the woman was advised of her personal risk estimate and specific strategies for her risk management were discussed.

The waiting list for the clinic rapidly grew. In the face of increasing demand for service only one appointment could be offered. The clinic was reorganized. Family history information is now collected in advance by post. Verification is undertaken and risk estimates are assigned as before. Women now receive information about their risk, clinical examination and risk management advice at a single visit. It was felt that the general educational component of the consultation process could equally well be presented on videotape. There were concerns that information given in advance of the clinic could cause anxiety and deter people

**Table 1** Video content – items rated by seven clinicians

| Items | Mean relevance rating (0–3) | Proportion giving item priority for inclusion |
|---|---|---|
| 1 Role of genes in the development of breast cancer | | |
| Breast cancer is common | 2.9 | 6/7 |
| Sporadic vs inherited breast cancer | 2.8 | 6/7 |
| Carrier vs non carrier | 2.4 | 4/7 |
| 2 Assessment of genetic risk from family history | | |
| Does the family have a relevant gene? | 2.4 | 4/7 |
| What is the chance a given individual has it? | 2.3 | 4/7 |
| Risk of cancer for carriers/non-carriers? | 2.1 | 4/7 |
| 3 What can be done to reduce risk? | | |
| Breast awareness | | |
| Self examination | 2.5 | 3/7 |
| Clinical examination | 2.3 | 1/7 |
| Screening | | |
| What is involved | 2.8 | 4/7 |
| Who should go | 2.6 | 3/7 |
| How often | 2.3 | 2/7 |
| Pros/cons | 2.6 | 4/7 |
| False alarms | 2.6 | 4/7 |
| Risk factors | | |
| Oral contraceptive pill[a] | 1.9 | 2/7 |
| Hormone replacement therapy | 1.8 | 2/7 |
| Diet[a] | 1.3 | 0/7 |
| Stress[a] | 1.3 | 0/7 |
| Benign breast disease[a] | 1.4 | 1/7 |
| Tamoxifen trial | | |
| How organized[a] | 1.6 | 2/7 |
| Pros/cons[a] | 1.8 | 2/7 |
| Preventive surgery | | |
| Breast[a] | 1.8 | 2/7 |
| Ovary[a] | 1.9 | 1/7 |
| 4 Related concerns | | |
| Risk counselling | 2.4 | 3/7 |
| Other health care screening | | |
| e.g. ovary | 2.4 | 3/7 |
| Prospects for genetic testing | 2.3 | 3/7 |

[a]Items not included in the video.

from attending. It was not clear whether the video would be more useful after the clinic to consolidate the information given.

We therefore set out to produce an educational videotape of broadcast quality to a give a general introduction, suitable for a wide lay audience, on the genetic basis of breast cancer and on strategies of breast cancer risk management, for example mammography screening. A complementary video was prepared at the same time for individuals at high risk. This was intended to provide more detailed information that was relevant to decision-making about whether or not to undergo genetic testing and to the subsequent choice of risk management strategy, for example chemoprevention and prophylactic surgery. Evaluation of the second video will be reported elsewhere.

The study to be reported here evaluated the use of the introductory video in the breast cancer family clinic by testing the following hypotheses. The use of videotaped information would:

1. reduce consultation time (when the video was used before the clinic);
2. increase the accuracy of women's estimate of their risk;
3. improve understanding of the basic concepts of breast cancer genetics and risk management;
4. not increase emotional distress;
5. increase satisfaction with the consultation for both the woman and the clinician;
6. promote family communication about the genetic issues covered in the video.

### METHODS

### Phase 1: video production

#### *Content*

Interim analysis of (as yet unpublished) psychological assessment data from the first cohort of women attending the clinic suggested a number of issues which should be covered by the video. These were summarized into a list (by AC). A geneticist (MS), a breast surgeon (EA) and an oncologist (JM) experienced in breast cancer risk counselling independently listed items of information that they felt an introductory video should convey. These lists were collated and reformatted after discussion to provide a single locally agreed list.

This list was circulated to clinicians running cancer family clinics in Aberdeen, Dundee and Glasgow and to the head of the

© Cancer Research Campaign 1998

NHS Clinical Genetics Service in SE Scotland. These seven clinicians were given a statement of the aim, intended use and target audience of the video. They were then asked to rate each item on the list on a four-point scale (0, not relevant; –3, very relevant) and to indicate (yes/no) whether the item was a priority for inclusion in the video. Items for inclusion had to have a mean rating > 1.5 and/or to be identified as a priority for inclusion by ≥ 3 of the seven staff making the ratings. Respondents were also invited to give qualitative feedback to inform the script-writing process and to identify any significant omissions.

A summary of the ratings awarded is given in Table 1. Items that were excluded on the basis of their ratings are marked with an asterisk. No significant omissions were noted.

The revised list of issues formed the basis of a script that was prepared by a professional script writer in collaboration with the authors. A draft script was circulated to the seven independent clinicians and revised in the light of their comments. The final version was circulated for approval before the video went into production.

The videos were produced by professional programme makers. Filming took place in the clinic with professional actors taking the part of the women counselled. The authors worked in close collaboration with the production team in editing the film and in the preparation of the commentary and graphics.

### *Professional evaluation of the videos*

Completed videos were sent to the five of the original seven clinicians who were available and to a member of the Institute of Medical Ethics. These six external reviewers used a four-point scale (1, poor; 2, adequate; 3, good; 4, very good) to rate the video on five attributes. All ratings had to be > 2 for the video to be used in the clinic. Mean ratings were as follows: coverage, 3.5; clarity, 3.7; presentation, 3.3; technical quality, 3.2; overall quality, 3.2. These ratings were sufficient to allow clinical evaluation to proceed.

### Phase 2: evaluation of video in the clinic

### *Participants*

A consecutive series of women newly referred to the breast cancer family clinic were invited by post to take part in the study.

### *Measures*

i. Sociodemographic data. Data were collected about the age, marital status, number of children, education and source of referral of all study participants.
ii. Time. The consultations with the geneticist and breast surgeon were timed.
iii. Risk assessment. Women were asked to select from 12 response categories which ratio they believed most closely represented their lifetime risk of developing breast cancer (Evans et al, 1993).
iv. Understanding of breast cancer genetics was assessed in two ways:
    a. Subjective assessment. Women were asked to rate on a four-point scale (1, not at all; –4, very well) how well they understood each of six issues relevant to breast cancer genetic risk. The issues were:
        1. how characteristics are inherited in families;
        2. how increased risk of breast cancer is passed on in families;
        3. whether or not your family has a genetically increased risk of breast cancer;
        4. the chance of you passing on an increased risk to your children;
        5. the pros and cons of mammograms for women under 50;
        6. the National Breast Cancer Screening Programme.
    b. Objective assessment. Four scenarios relating to breast cancer genetics were devised with multiple choice questions to assess women's understanding of concepts covered by the video. Key elements of those scenarios are summarized in Table 2. There were 21 scorable items in all.
v. Emotional distress was assessed using two methods:
    a. Spielberger State–Trait Anxiety Inventory (Spielberger et al, 1983) was used to assess anxiety proneness (trait) and anxiety levels at the time of the assessment (state).
    b. GHO-30 (Goldberg and Williams, 1988) was used to screen for clinically significant psychological disorder.
vi. Satisfaction with clinic. Ratings were obtained from the women attending the clinic and from clinicians.
    a. Women rated each of eight items about the information received at the clinic on a seven-point scale (1, not at all satisfied; –7, very satisfied).
    b. Both clinicians (geneticist and breast surgeon) rated five items concerning their assessment of the consultation, each on a seven point scale, for example proportion of time spent on general education/individual specific information; the woman's participation in the consultation; her understanding as assessed by clinician; clinician's satisfaction with consultation.
vii. Use of video. Women were asked to report whether they watched the video (in full), how many times, with whom and whether they had discussed genetic issues with family or friends. They were invited to indicate whether they had found the video confusing or upsetting and whether information which they had hoped for was not covered by the video.

### *Procedure*

Ethical approval for this study was obtained from the Regional Ethics Committee.

Women newly referred to the Breast Cancer Family Clinic were sent information about the video evaluation study with their clinic appointment 4 weeks before the appointment date. All women

**Table 2** Objective assessment of understanding – summary of scenarios

1. A woman of 45 whose mother recently died of breast cancer has been told there is no evidence of inherited breast cancer in her family (eight items about her chances of developing breast cancer and risk management options).
2. In families in which there is inherited susceptibility to breast cancer (seven items about who can pass on inherited susceptibility to whom).
3. A woman with breast cancer has four daughters. The eldest develops breast cancer and the family are confirmed as being at genetically increased risk (two items on risk to remaining sisters).
4. In a family with inherited susceptibility (four items on carriers/non-carriers: chances of passing on inherited susceptibility and developing cancer themselves).

© Cancer Research Campaign 1998

**Figure 1** Assessment procedure. The measures administered at each assessment are referred to by the numbers used in Measures referred to this clinic are routinely asked to complete a set of psychometric assessment questionnaires before they attend. For the purposes of this study they were invited to return baseline questionnaires and informed consent forms (in a stamped addressed envelope provided) within 2 weeks if they wished to take part in the video evaluation. Those agreeing to participate were randomized to receive the video before or after their clinic consultation. Those randomized to the 'Video Before' group were sent a copy of the video approximately 10 days before the consultation. The 'Video After' group received their copy of the video to take home with them after the clinic.

The clinic consultation proceeded as normal. Each woman saw a consultant geneticist to discuss their individual risk and a consultant breast surgeon to discuss risk management. Both these clinicians were blinded to the randomization. The clinicians noted the duration of each consultation and rated their assessment of it. The women completed the psychometric assessments again immediately after their consultation at the clinic and at postal follow-up 1 month later. A flow chart listing the measures administered at each assessment is shown in Figure 1.

### *Statistical analysis*

Statistical comparisons were made by chi-squared tests for nominal variables, Mantel–Haenszel tests for trend for ordinal variables and Mann–Whitney tests for quantitative variables. The correlation between educational level and scores for objective understanding of genetic concepts was calculated using Spearman's rank correlation. Comparison of the objective knowledge of the two randomized groups was undertaken by analysis of covariance to adjust for the imbalance in educational level across the two groups.

### RESULTS

### The sample

One hundred and fifty-nine women were invited to take part in the study, 15 (19%) refused. The reasons for refusal varied. Six women were clearly anxious about the study: two said they were too shy to give opinions, one reported difficulty absorbing information that she thought would make it difficult for her to take part; one thought the video would put her off attending the clinic and another that the study would be a source of worry. The sixth woman simply said she felt unsure. Another misunderstood what she was being asked to do and replied she was too fat to take part. Of the remaining eight, two women had no video player, three were too busy, two were about to go on holiday and the eighth refused because as a breast care nurse she felt her responses would be atypical.

Of 144 women randomized, eight rearranged the time of their clinic appointment to fall outwith the study period. Only one of them had received the video. Eight women failed to attend the clinic without explanation of whom five belonged to the Video Before and three to the Video After group. One hundred and twenty-eight (80%) women agreed to take part in the study and attended the clinic. Sixty-six were randomized to receive the Video Before the consultation of whom 53 (80%) returned postal follow-up data. Sixty-two received the Video After their consultation, of whom 42 (68%) returned postal follow-up data. No significant differences were found in the baseline measures of those who did and did not return postal follow-up data.

The mean age of the women in the study was 39 years (s.d. = 8 years). There was no age difference between the two groups. Women in the Video After group were more likely to be divorced (19% vs 4%). There was no significant difference in family size or number of daughters between the women in the two groups (mean family size = 1.6 children, s.d. = 1.2; mean number of daughters = 0.6, s.d. = 0.7). Information about educational attainment was available for all but three of the Video After group. The sample as a whole were highly educated with 60 out of 125 having had some tertiary education after age 18. The Video After group were better educated with more women with a university education (37% vs 18%) and fewer educated only to age 16 (27% vs 41%). Twenty four per cent of the Video Before and 30% of the Video After group had been referred from another hospital clinic. One woman in each group had been referred from another genetic clinic. The remaining women were referred by their general practioner.

### Duration of consultation

The Video Before group spent significantly less time with the breast surgeon (Video Before: mean = 11.8 min ± 5.4 vs Video

© Cancer Research Campaign 1998

Table 3 Ratings of subjective understanding reported by each group after clinic consultation

| Item group | n | Not at all | A little | Quite well | Very well | $\chi^2$ P (d.f. = 1) |
|---|---|---|---|---|---|---|
| 1. Video Before | 61 | – | 18% | 64% | 18% | $\chi^2 = 11.28$ |
| Video After | 60 | 5% | 43% | 42% | 10% | $P < 0.001$ |
| 2. Video Before | 62 | – | 11% | 58% | 31% | $\chi^2 = 15.89$ |
| Video After | 60 | 2% | 32% | 60% | 7% | $P < 0.001$ |
| 3. Video Before | 62 | 2% | 13% | 55% | 31% | $\chi^2 = 1.62$ |
| Video After | 60 | 2% | 20% | 57% | 21% | NS |
| 4. Video Before | 61 | 3% | 16% | 44% | 36% | $\chi^2 = 11.92$ |
| Video After | 58 | 7% | 26% | 64% | 3% | $P < 0.001$ |
| 5. Video Before | 62 | 2% | 13% | 52% | 34% | $\chi^2 = 6.18$ |
| Video After | 60 | 2% | 28% | 53% | 17% | $P < 0.05$ |
| 6. Video Before | 62 | 5% | 14% | 50% | 31% | $\chi^2 = 5.70$ |
| Video After | 60 | – | 33% | 58% | 8% | $P < 0.05$ |

Items 1–6 refer to the issues specified in Measures of the text under the heading iv Understanding of breast cancer genetics

After: mean = 14.6 min ± 7.2; $Z = 1.99$, $P < 0.05$) but their consultation time with the geneticist was not significantly shorter (mean time = 12.3 min ± 6.0 vs Video After: mean time = 13.1 min ± 6.3).

### Risk assessment

There was no significant difference between the two groups at baseline in the accuracy of their estimate of their own risk of developing breast cancer. Fifty-nine per cent of women in each group were within twofold of the counselled risk, 27% underestimated by ≥ × 0.5 and 14% overestimated by ≥ × 2. There was no significant difference between the two groups in the accuracy of their risk estimates immediately after the consultation. Both groups were more accurate after counselling: 81% were within twofold of the risk given by the counsellor, 17% underestimated by ≥ 0.5 and 2% overestimated by ≥ × 2 in each group. Postal follow-up data were available for 50 of the Video Before group and 39 of the Video After group. The Video Before group retained the level of accuracy shown at clinic at postal follow-up. The Video After group were significantly more likely to underestimate in their personal risk estimate at postal follow-up ($\chi^2 = 4.62$, d.f. = 1, $P < 0.05$). A total of 38% underestimated by ≥ 0.5 compared with 18% of the Video Before group.

### Understanding of breast cancer genetic risk information

### *Subjective assessment of understanding*

Ratings for individual items and the summed scores for subjective understanding were compared between the groups. At baseline there was no significant difference in understanding between the groups on any of the six categories of information or overall. After the clinic consultation the Video Before group reported significantly better understanding of five of the six items (Table 3) and hence a better overall score ($Z = 3.65$, d.f. = 1, $P < 0.001$).

At this point the Video Before group had seen the video. The Video After group had had counselling alone. The issues itemized in Measures are referred to by number in Table 3. At postal follow-up there was again no significant difference between the groups.

### *Objective assessment*

Responses to individual items were compared between the two groups. Correct responses were summed to give a total score for objective understanding for each group at each assessment point. The study was designed to compare objective understanding after the clinic consultation ± video. Understanding was assessed twice only: after the clinic consultation and at postal follow-up. At the first of these time points the Video Before group had information from the video and from the consultation. The Video After group had the information from the consultation alone. By the second assessment, both groups had information from both sources.

The Video Before group obtained higher scores for understanding ($Z = 2.91$, d.f. = 1, $P < 0.01$) and in particular had a significantly higher proportion of correct responses to five of the items. The scenarios to which these items refer can be identified by number with reference to Table 2. The items that discriminated between the groups were:

***Scenario 1. Should this woman join the National Screening programme now?***

The Video Before group were more likely to understand that a woman of 45 years is too young for this programme (37% vs 20%; $\chi^2 = 4.13$, d.f. = 1, $P < 0.05$).

***Scenario 2a. Can any woman in such a family pass on inherited risk to her children?***

The Video Before group were more likely to understand that not all women in the family will have inherited the increased risk (45% vs 25%; $\chi^2 = 4.17$, d.f. = 1, $P < 0.05$).

***Scenario 2b. If a parent has inherited a gene causing increased susceptibility to breast cancer to which of their children are they likely to pass on that gene?***

The Video Before group were significantly more likely to understand that the gene was likely to be passed to half of all the children i.e. both sexes (39% vs 15%; $\chi^2 = 7.53$, d.f. = 1, $P < 0.01$).

***Scenario 3. Another sister in this family develops breast cancer. Does that influence the risk (of developing breast cancer) for the remaining sisters?***

The Video Before group were more likely to understand that their risk was unchanged (61% vs 42%; $\chi^2 = 3.95$, d.f. = 1, $P < 0.05$)

© Cancer Research Campaign 1998

***Scenario 4. In such a family could a woman who has not inherited the genetic susceptibility still pass on an increased risk to her daughters?***

The Video Before group were more likely to understand that the woman could not pass on a gene which she herself has not inherited (86% vs 62%; $\chi^2 = 7.51$, d.f. = 1, $P < 0.01$).

There were no significant differences between the groups in their knowledge at postal follow-up. A correlation was observed between educational level and objective understanding at post-clinic follow-up (Spearman's rho = 0.33, $P < 0.01$). Given the imbalance in educational level between the two groups an analysis of covariance was undertaken that confirmed that after adjusting for educational level there was no significant difference in objective understanding between the two groups by the time both had had counselling and seen the video ($t = 0.34$).

### Emotional Distress

### Anxiety

There was no significant difference between the groups in anxiety proneness. The mean trait anxiety score for the Video Before group was 40 (s.d. = 10) and for the Video After group was 42 (s.d. = 10). The mean state anxiety scores were: Video Before group: baseline, 35 (s.d. = 11); clinic, 34 (s.d. = 10); postal follow-up, 32 (s.d. = 9). Video After group: baseline, 38 (s.d. = 14); clinic, 34 (s.d. = 10); postal follow-up, 35 (s.d. = 13).

### GHQ-30

At baseline the mean GHQ score and proportion of women scoring above the cut-off for case-level distress (i.e. > 4) was higher in the Video After group but the differences were not statistically significant. There were no significant differences in group mean scores across assessment points and no evidence of an association between exposure to the video and increased distress (Table 4).

### Satisfaction with clinic

Women in the Video Before group gave a higher proportion of high ratings (6, 7) for all items. They were significantly more satisfied than the Video After group with information given about genetics (92% vs 73%; $\chi^2 = 6.14$, d.f. = 1, $P < 0.05$); breast cancer (73% vs 62%; $\chi^2 = 4.51$, d.f. = 1, $P < 0.05$) and access to breast screening (87% vs 72%; $\chi^2 = 4.27$, d.f. = 1, $P < 0.05$). Clinicians expressed no difference in their ratings of the consultation between the two groups.

**Table 4** GHQ scores

| | Video Before | Video After |
|---|---|---|
| Baseline | | |
| *n* | 66 | 62 |
| Mean score (SD) | 3.9 (5.8) | 5.8 (7.1) |
| Number of cases (%) | 19 (29%) | 25 (40%) |
| Clinic | | |
| *n* | 66 | 61 |
| Mean score (s.d.) | 3.6 (6.0) | 5.7 (7.9) |
| Mean change from baseline | – 0.3 (4.5) | –0.2 (6.3) |
| Number of cases (%) | 15 (23%) | 22 (36%) |
| Became case | 4 (9%) | 6 (16%) |
| Ceased to be case | 8 (42%) | 9 (36%) |
| Postal follow-up: | | |
| *n* | 53 | 42 |
| Mean score (s.d.) | 3.1 (5.7) | 3.9 (7.0) |
| Number of cases | 10 (19%) | 12 (29%) |
| Change from clinic: | | |
| Became case | 3 (6%) | 1 (3%) |
| Ceased to be case | 7 (47%) | 4 (18%) |

### Use of video and family discussion

### Video Before group

A total of 94% of the Video Before group watched the video at least once from start to finish. Only 21 of the 66 women watched it twice or more. No significant differences were observed in the objectively assessed understanding of those who watched the video once vs more times. Most (65%) watched it alone. Those who watched it with someone else were most likely to watch it with their partners ($n$ = 16). Whether or not the partner saw the video, 30 women discussed the video with their partner. The numbers of women who watched the video with relatives were: mothers, 5; fathers, 1; sisters, 3; brothers, 0; daughters, 1; sons, 0. The numbers who discussed the video with relatives were: mothers, 7, fathers, 1; brothers, 1; daughters, 3; sons, 2. Five women watched the video with a friend and 11 discussed it with a friend.

Fifty women (76%) reported that the video offered them new information. Three women reported finding some information given on the video unclear or confusing about: whether cancer at other body sites could be inherited; the basis for the association between breast and ovarian cancer risk and the risk of breast cancer occurring sporadically in a family with inherited susceptibility. Eight women identified information they had hoped to get that was not on the video: three wanted information about early detection of cancer, for example 'What does a cancerous breast look like?'; two wanted more information about genetic testing; two wanted to know more about cancer treatment and research and one wanted to know about other causal factors. Six women reported finding some of the information given upsetting. For three, the video triggered memories of relatives who had died of breast cancer; two mentioned increased awareness of the risk to themselves and their children and one said the topic was inevitably a source of general concern.

Ratings of satisfaction with the use of the video as a way of giving information were returned by 59 women. Fifty-two of them rated their satisfaction 6 or 7 on a seven-point scale (ranging from 1, not at all satisfied to 7, very satisfied). Only one woman gave a rating at the mid-point of the scale (neutral) and there were no ratings on the negative axis of the scale. Women who watched the video more than once were no more accurate in their personal risk estimate after counselling than women who watched the video once only. Women were offered the opportunity to keep their copy of the video, but because of the limited number of copies available they were asked to pay its cost price (£2.50). Only three women elected to keep the video and they did watch it again after the clinic but the numbers were, therefore, too small for further analysis of the impact on understanding.

### Video After group

A total of 42 women (68%) returned the postal follow-up data 4 weeks after the clinic. Forty-one of them watched the video at least once. A total of 66% of respondents watched the video alone. Those who watched it with someone else were most likely to watch it with their partners ($n$ = 11) and 18 women discussed the

© Cancer Research Campaign 1998

video with their partners. The number of women watching the video with relatives were: mothers, 5; fathers, 1; sisters, 9; brothers, 1; daughters, 2; sons, 0. The number discussing the video with these relatives were: mothers, 8; fathers, 4; sisters, 13; brothers, 4; daughters, 3; sons, 2. Four women watched the video with a friend and nine discussed it with a friend. In this group one woman also showed the video to her GP.

A total of 25 out of 41 respondents (61%) who viewed the video for the first time after their clinic consultation said it gave them information they did not have before. Only four of the 42 women in the Video After group found any of the information unclear or confusing: concerning the relationship between breast and ovarian cancer risk; about the male role in transmitting susceptibility to breast cancer; the age at which women should undergo mammographic screening and how risk estimates are expressed. Two women said the confusion arose in trying to reconcile what they understood from the clinic with what they saw on the video.

Ten of the 42 women found information for which they hoped was not on the video. Two wanted information about the male role in transmitting susceptibility to breast cancer; five wanted more detailed information about the relevant genes ($n = 2$); about the age at which genetic breast cancer is likely to develop and whether the type of tumour that develops is different in sporadic vs inherited breast cancer ($n = 2$). Three women wanted more information about risk management including breast self-examination ($n = 1$) and diet ($n = 1$).

Two women reported some distress associated with the video. One said 'no-one wants to think about developing cancer', the other was distressed to realize that genetic testing would not automatically be available.

### DISCUSSION

Women referred to this breast cancer family clinic welcomed the opportunity to take part in this study with a response rate of 89%. There was no evidence that seeing the Video Before the clinic deterred women from attending. Compliance with the study was better among the Video Before group who also expressed more satisfaction with the clinic.

The production of the video was supported by the NHS Research and Development programme in cancer. It was therefore important that the results of the study should be generalizable across the NHS. The views of experienced clinicians across four Scottish regions informed the video content. Their feedback on the finished product confirmed its applicability at least across the Scottish clinics.

The women in this study were better educated than the norm for the general population. This is a common bias in health screening samples (Rimer et al, 1996). Videotaped information viewed before the clinic improved understanding of relevant concepts as assessed after the consultation even though the Video Before group were less well educated than the Video After group, i.e. with half the number of university graduates (12 vs 22). Repeated viewing of the video by the small numbers in this study was not associated with a significant improvement in understanding relative to that achieved after a single viewing. Even so, in this well-educated sample, as a whole the prevalence of misunderstandings elucidated by this study was salutary. Further studies will be needed to determine the most cost-effective ways to communicate such complex probabilistic and emotive information to members of the general public who are less well educated.

The first hypothesis was confirmed. The amount of consultation time saved by prior use of the video was statistically but not clinically significant. The consultation time offered is already short, given the volume and complexity of information to be conveyed. It may be that by offering general educational information in advance, a greater proportion of the consultation can be spent on the woman's concerns but the clinicians' ratings of the consultation used in this study were not sufficiently sensitive to pick this up. Investigation of the counselling process, which is urgently needed, requires more sophisticated methodology including recording and independent rating of the interaction.

In contrast to the US data (Lerman et al, 1995) a minority of women (14%) had grossly exaggerated preconceptions of their risk and these appeared responsive to counselling. Similar data have been reported from Manchester (Evans et al, 1994) and in our earlier study (Cull et al, 1995). Evidence to support the second hypothesis was equivocal. Prior use of the video appeared to confer no advantage in women's ability to report their risk estimate immediately after counselling but the Video Before group gave more accurate risk estimates than the Video After group at one month follow-up. Prior information may have enhanced understanding and hence retention of the risk estimate given at the clinic. These data must be interpreted with caution given the differential response rate between the two groups at follow-up but this interpretation is supported by evidence of improved understanding of genetic issues reported and exhibited by the Video Before group.

Prior use of the video appeared to confer an advantage over counselling alone in terms of the women's subjective assessment of their understanding and their responses to the objective measure used. At 1 month follow-up, when the Video After group had seen the video this advantage was lost. This supports the hypothesis that the video was responsible for improving understanding.

It might have been desirable to have had an objective measure of the women's understanding at baseline. There is some sensitivity about the use of a measure that highlights misunderstanding and that may thus provoke non-compliance as a defence against appearing ignorant. It was, therefore, felt inappropriate to administer the objective measure for the first time postally at baseline. The design adopted which did not assess objective understanding until after the consultation, then repeated the assessment at postal follow up, gave an opportunity for this measure to be introduced as an evaluation of the information given rather than of the woman's understanding per se.

The persistent misunderstandings demonstrated on objective assessment were revealing. The first scenario concerned a woman of 45, who had been counselled that she was not at significantly increased risk but with a history of the recent loss of her mother (presumably at age > 60) from breast cancer. The majority of respondents in both groups felt this woman should seek a second opinion about her risk, ask her GP for regular breast examination and seek annual mammograms. The second scenario showed that a minority of respondents understood that if a parent carries a gene causing susceptibility to breast cancer they may pass that to children of either sex but on average only half of their children will inherit that gene. There was particular confusion over the concept of men as carriers of the gene. In scenario three, which referred to a family identified as having the relevant gene, only half of this sample understood that the risk for each individual of being found to carry the gene was 1 in 2. The fourth scenario further highlighted confusion about what non-carriers (in a family with inherited susceptibility) can/cannot pass to their offspring.

 © Cancer Research Campaign 1998

Using a well-validated screening measure – GHQ-30 – there was no evidence that in most women the use of videotaped information caused significant psychological distress. A measure of the extent of cancer-related worry, for example Kash et al (1992), might have been more informative than the Spielberger measure of generalized anxiety which we used. A minority of women did report some distress associated with viewing the video. Open-ended questioning revealed that this reflected recollection of family bereavements and anxiety about personal risk that were seen as integral to the process of seeking information about a positive family history and not exclusively triggered by the video.

The rating scales used, demonstrated a significant increase in satisfaction with the clinic expressed by those who received the video in advance. However, the clinicians in this busy clinic were appropriately blinded to the allocation of videos and conducted all consultations routinely. This suggests that the impact of the video could be increased in clinical use by explicitly integrating it into the consultation process, for example advising women to use the video to generate questions to put to the clinician and referring them back to the video for consolidation of the information given at clinic.

The assessment method used to assess family communication about the video was weak without corresponding data about the numbers and availability of the relevant family members to aid interpretation of the information given. Women viewing the video before the clinic tended to discuss the relevant issues more with their partners and friends than with blood relatives. They appear more likely to involve female than male relatives.

Omissions in the coverage of the video noted by a minority of women all concerned more detailed information that had been incorporated in the second video, which was designed for women confirmed to be at significantly increased risk, this included the issues in BRCA1 testing and reviews, the role of screening, chemoprevention and prophylactic surgery as risk management strategies.

Our study supports the value of videotape as a method of giving general information to women about genetic risk and breast cancer screening before their clinic appointment. Although the data confirm that videotaped information can improve understanding without causing distress, the study also underlines the need for counsellors to check for misunderstandings about key concepts. Women seeking this information may have recent experience or reawakened memories of cancer in the family that are distressing and clinicians need to remain alert to complicated bereavement reactions and levels of psychological disturbance that warrant specialist referral. In the context of a family history clinic, this video should be used as an aid to, not a substitute for, improved communication about inherited susceptibility to breast cancer.

### ACKNOWLEDGEMENTS

The authors wish to thank: Morag Fullerton, the author of the video script; Mrs Joyce Campbell and Mrs Elizabeth Smyth for their contribution to the project; Dr Kenneth Boyd, Dr Rosemarie Davidson, Dr John Dewar, Dr Fiona Douglas, Dr Helen Gregory, Dr Neva Haites, Dr Mary Porteous and Mr Paul Preece for their work in reviewing the video development. The project was supported by funding from the NHS R&D (Cancer) Programme and the Imperial Cancer Research Fund. Copies of the video are available on request from the first author.

### REFERENCES

Cull A, Anderson E, Mackay J, Smyth E, Steel CM and Prescott R (1995) Risk perception and distress among women attending a breast cancer family clinic. *Eur J Cancer* **31A**: 277

Evans DGR, Burnell LD, Hopwood P and Howell A (1993) Perception of risk in women with a family history of breast cancer. *Br J Cancer* **67**: 612–614

Evans DGR, Blair V, Greenhalgh R, Hopwood P and Howell A (1994) The impact of genetic counselling on risk perception in women with a family history of breast cancer. *Br J Cancer* **70**: 934–938

Goldberg D and Williams P (1988) *A Users Guide to the GHQ*. NFER Nelson Publishing: Berkshire UK

Hallowell N, Murton F, Stratham H, Green JM and Richards MPM (1997) Women's need for information before attending genetic counselling for familial breast or ovarian cancer: A questionnaire interview and observational study. *Br Med J* **314**: 281–283.

Kash KM, Holland JC, Harper MS and Miller DG (1992) Psychological distress and surveillance behaviours of women with a family history of breast cancer. *J Natl Cancer Inst* **84**: 24–30

Lerman C, Lustbader E, Rimer B, Daly M, Miller S, Sands C and Balshem A (1995) Effects of Individualised Breast Cancer Risk Counseling: A Randomised Trial. *J Natl Cancer Inst.* **87**: 286–292.

Lloyd S, Watson M, Waites B, Meyer L, Eeles R, Ebbs S and Tylee A (1996) Study of risk perception, psychological morbidity and health beliefs in women attending for genetic counselling. *Br J Cancer* **74**: 482–487

Rimer BK, Schildkraut JM, Lerman C, Lin TH and Audrain J (1996) Participation in a women's breast cancer risk counselling trial. Who participates? Who declines? *Cancer* **77**: 2348–2355

Schapira MM, Meade C and Nattinger AB (1997) Enhanced decision making: the use of videotaped decision aid for patients with prostate cancer. *Patient Education Counselling* **30**: 119–127

Spielberger CD, Gorusch RL and Luchene RE (1983) *State Trait Anxiety Inventory: Manual*. Consulting Psychologists Press: Palo Alto, CA